# The amygdala and the pursuit of future rewards

**DOI:** 10.3389/fnins.2024.1517231

**Published:** 2025-01-22

**Authors:** S. Tobias Johnson, Fabian Grabenhorst

**Affiliations:** Department of Experimental Psychology, University of Oxford, Oxford, United Kingdom

**Keywords:** amygdala, reward, temporal discounting, effort, depression, goal pursuit, planning

## Abstract

The successful pursuit of future rewards requires forming an internal goal, followed by planning, decision-making, and progress-tracking over multiple steps. The initial step—forming goals and the plans for obtaining them—involves the subjective valuation of an anticipated reward, considering both the reward’s properties and associated delay and physical-effort costs. Recent findings indicate individuals similarly evaluate cognitive effort over time (Johnson and Most, 2023). Success and failure in these processes have been linked to differential life outcomes and psychiatric conditions. Here we review evidence from single-neuron recordings and neuroimaging studies that implicate the amygdala—a brain structure long associated with cue-reactivity and emotion—in decision-making and the planned pursuit of future rewards (Grabenhorst et al., 2012, 2016, 2019, 2023;Hernadi et al., 2015;Zangemeister et al., 2016). The main findings are that, in behavioral tasks in which future rewards can be pursued through planning and stepwise decision-making, amygdala neurons prospectively encode the value of anticipated rewards and related behavioral plans. Moreover, amygdala neurons predict the stepwise choices to pursue these rewards, signal progress toward goals, and distinguish internally generated (i.e., self-determined) choices from externally imposed actions. Importantly, amygdala neurons integrate the subjective value of a future reward with delay and effort costs inherent in pursuing it. This neural evidence identifies three key computations of the primate amygdala that underlie the pursuit of future rewards: (1) forming a self-determined internal goal based on subjective reward-cost valuations, (2) defining a behavioral plan for obtaining the goal, (3) executing this plan through stepwise decision-making and progress-tracking. Based on this framework, we suggest that amygdala neurons constitute vulnerabilities for dysfunction that contribute to maladaptive reward pursuit in psychiatric and behavioral conditions. Consequently, amygdala neurons may also represent potential targets for behavioral-change interventions that aim to improve individual decision-making.

## Introduction

The best rewards are often distant and can only be obtained by prospective (i.e., future-oriented) valuation of goals, planning and decision-making over several steps, and the tracking of progress toward a goal. Some future rewards can be obtained over shorter timescales, such as planning a restaurant visit in the evening to consume a desired food. However, many rewards are much more distant and require planning and persistence over longer timescales, including economic saving, achieving physical or mental health outcomes, pursuing an academic degree, or finding one’s ideal partner. What neural mechanisms underlie the pursuit of future rewards? What information is encoded by neurons in the brain’s reward system when an individual forms a goal and pursues it through planning and decision-making? We argue that investigating these neurophysiological mechanisms can validate or challenge psychological conceptions of goal pursuit and advance understanding of psychiatric and behavioral conditions in which this the pursuit of future rewards is impaired.

Psychological and behavioral-economic theories identify two key principles for planned reward-guided behaviors (Miller et al., 1960; Gollwitzer, 1993; Dickinson and Balleine, 1994; Sutton and Barto, 1998; Benhabib and Bisin, 2005; Berkman and Lieberman, 2009): the initial formation of a goal based on subjective reward and cost valuations, and the pursuit of this goal through planning, decision-making and progress-tracking. The valuation step is complex, as it requires an individual to consider the rewarding properties of a potential goal (e.g., its magnitude, desirable sensory qualities, relevance to one’s long-term values) and its associated costs (e.g., the delay, physical and cognitive effort required for obtaining it), and evaluating these factors based on subjective preferences and aversions.

Success and failure in the component process underlying the pursuit of future rewards have been linked to differential health outcomes, well-being, and specific psychiatric and behavioral conditions (Bechara, 2005; Casey et al., 2011; Bickel et al., 2012; Korn et al., 2014; Gillan et al., 2020). Accordingly, behavioral-change interventions to improve physical and mental health target the formation of resilient internal goals and the commitment to pursuing them (Gollwitzer and Schaal, 1998; Diamond and Lee, 2011; Belanger-Gravel et al., 2013; Duckworth et al., 2013; Michie et al., 2013; Sheeran et al., 2013; Scholten et al., 2019). For example, ‘mental contrasting’ involves imagining both the positive outcomes of a distant goal and possible obstacles to achieving the goal (Duckworth et al., 2013). Such interventions are designed to strengthen an individual’s capacities for successful pursuit of long-term goals. These capacities are constrained by the effort and delay involved in goal achievement, as well as inconsistencies in evaluating these cost factors (Ainslie and Herrnstein, 1981; Johnson and Most, 2023).

Here, we review recent advances in understanding the neurophysiological basis for the pursuit of future rewards in one of the key brain areas implicated in reward and decision-making: the primate amygdala. We first summarize the amygdala’s anatomical organization and its functions. We then discuss psychological and behavioral-economic conceptions of goal formation and planned behavior, and introduce evidence on delay and effort cost valuation. These considerations identify some of the key processes that might be encoded by neural systems during planned reward-directed behavior. Next, we review findings from single-neuron recordings in the amygdala of monkeys performing a reward-based economic saving task, and from related human neuroimaging studies. These studies show that amygdala neurons encode internally determined goals for future rewards and the decisions and plans for obtaining them. Finally, we discuss how prospective amygdala signals may constitute vulnerabilities for dysfunction in psychiatric and behavioral conditions, and targets for interventions aiming for behavioral change. Table 1 summarizes our neuronal framework for the pursuit of future rewards, based on identified neural signals in the primate amygdala, and its implications for understanding related dysfunction and for conceptualizing behavioral interventions in terms of neural mechanisms.

**Table 1:** Neuronal framework for the pursuit of future rewards, vulnerabilities for dysfunction, and targets for behavioral interventions affecting neuronal signals.

Neuronal signals in primate amygdala	Properties of neural signals and proposed functions	Potential vulnerability, intervention target
Sequence value (Hernadi et al., 2015; Zangemeister et al., 2016)	Properties: reflects subjective goal valuation based on future reward attributes, delay and effort costs; often specific for self-determined behavior; fluctuates with performance errors Functions: initial goal selection based on value; guides plan formation and reward expectation, arousal, attention during goal pursuit	Vulnerability: failure to integrate reward attributes, delay and effort costs based on subjective value; goals may be unrealistic, low-valued, or overly ambitious Intervention target: direct attention away from costs of distant goals toward their rewarding components to strengthen resilience of sequence-value signals and reduce likelihood of temporary-preference formation; e.g., enjoyment of cognitive effort may buffer against dynamic inconsistency (Johnson and Most, 2023)
Sequence length (Hernadi et al., 2015; Zangemeister et al., 2016)	Properties: reflects length of planned choice sequence; often specific for self-determined behavior; fluctuates with performance errors Functions: translates goal into behavioral plan to achieve the goal, guides progress-tracking and step-by-step choices	Vulnerability: failure to form appropriate behavioral plan to obtain selected goal; breakdown of plan signal during goal pursuit Intervention target: division of long-term goals into sub-goals, thereby promote sequence-length neurons to encode shorter-term plans to achieve sub-goals; supportive strategies at times of error could stabilize sequence-length encoding
Save-spend choice (Grabenhorst et al., 2012)	Properties: reflects save-spend choice at choice points within a planned behavioral sequence; often specific for self-determined behavior Functions: guides current-step choice (immediate vs. distant reward) in accordance with plan and final goal	Vulnerability: failure to select single-step choice in alignment with current goal and plan Intervention target: change single-step choices through support at critical choice points; top-down influences based on more resilient goals, mental contrasting and other goal setting strategies solidify final goals values to bias save-choice neurons, reducing likelihood of premature goal abandonment
Sequence progress (Grabenhorst et al., 2016)	Properties: reflects current progress in choice sequence toward final goal; adapts to planned final sequence length; often specific for self-determined behavior; fluctuates with performance errors Functions: monitoring of progress toward goal to guide step-by-step choices	Vulnerability: breakdown of progress-tracking could lead to premature goal abandonment Intervention target: develop methods of tracking progress toward distant goals such as regular performance measurements, or daily trackers to stabilize progress-tracking and ensure progress is scaled to reflect final reward goal to prevent goal abandonment; lower the gain (i.e., slope) of amygdala progress signals to enhance ‘patience’; supportive strategies at times of error could stabilize sequence-progress encoding

## The primate amygdala: overview of structure and function

**Figure 1:**
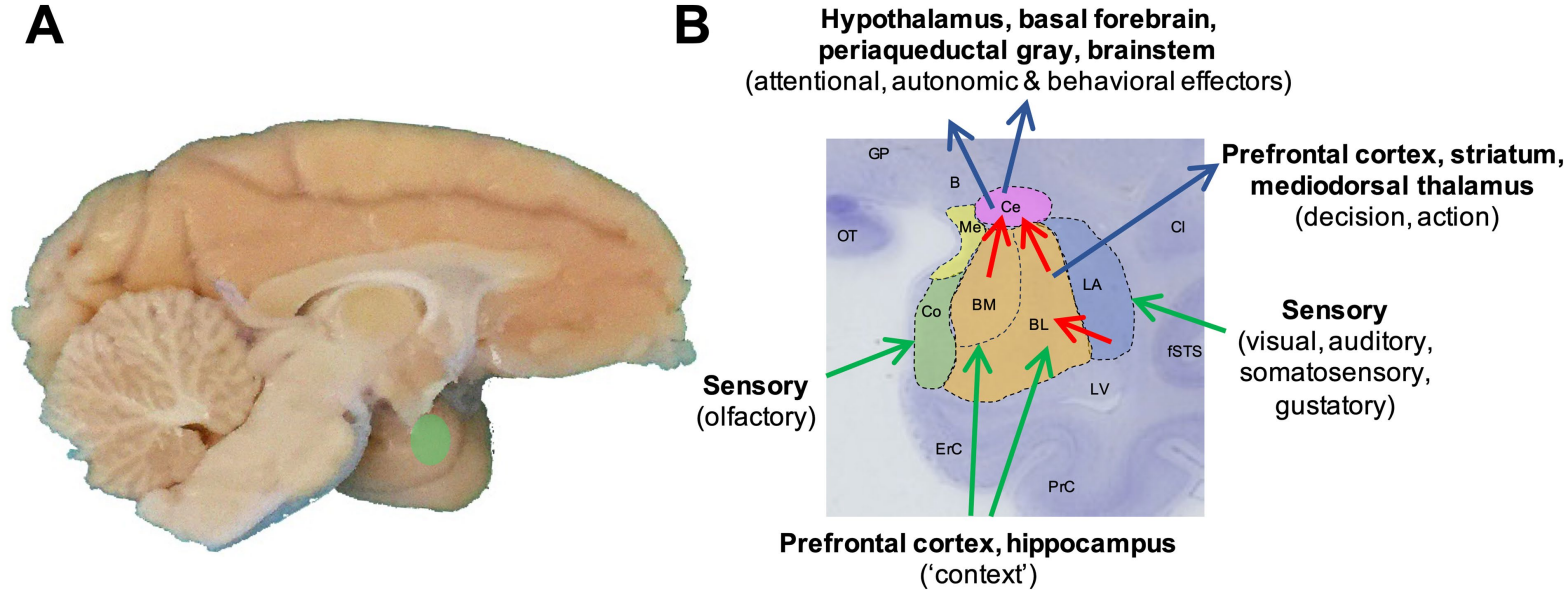
Primate amygdala anatomy and connectivity. **(A)** Location of the amygdala in the anterior-medial part of the temporal lobe of the primate brain (Macaca mulatta). **(B)** Schematic overlaid on a cresyl-violet stained macaque coronal brain slice illustrating major amygdala nuclear subdivisions considered in this review, and some of their main input/output connections with simplified functional descriptions. Note that the sensory connections are typically bidirectional (Price, 2003). Blue: lateral nucleus (LA); orange: basolateral (BL) and basomedial nuclei (BM); magenta: centromedial nucleus (Ce); green: cortical nucleus (Co); yellow: medial nuclei (Me). B: basal nucleus of Meynert; Cl: claustrum; ErC: entorhinal cortex; fSTS: fundus of the superior temporal sulcus; GP: globus pallidus; LV: lateral ventricle; OT: optic tract; PrC: perirhinal cortex. Nomenclature based on Paxinos et al. (2000).

The amygdala, a cell complex located in the anterior-medial temporal lobe (Figure 1A), has long been associated with mediating emotional reactions to sensory cues (Rolls, 2000; Baxter and Murray, 2002; Cardinal et al., 2002; Maren and Quirk, 2004; Balleine and Killcross, 2006; Murray, 2007; Ghods-Sharifi et al., 2009; Morrison and Salzman, 2010; Johansen et al., 2011; Janak and Tye, 2015; Gothard, 2020; Pujara et al., 2022). However, recent findings also implicate primate amygdala neurons in more complex cognitive functions, including the pursuit of future rewards through economic, value-based decision-making and planning (Grabenhorst et al., 2012; Hernadi et al., 2015; Grabenhorst et al., 2016; Grabenhorst et al., 2019; Grabenhorst and Schultz, 2021; Grabenhorst et al., 2023).

The primate (including human) amygdala is composed of several nuclei that vary in cell types, connections, and functions [for detailed reviews, see (Price et al., 1987; Price, 2003; Schumann et al., 2016)]. In a simplified view (Figure 1B), the lateral nucleus can be conceptualized as the amygdala’s entry point for sensory signals and storage site for stimulus-reinforcer associations: it receives inputs from all sensory systems, including particularly rich visual inputs from the inferior temporal cortex (Stefanacci and Amaral, 2002), and its neurons show potentiated responses to sensory stimuli that have been associated with reward or punishment (Sah et al., 2003; Johansen et al., 2011; Duvarci and Pare, 2014). The lateral nucleus projects to the basolateral and basomedial nuclei, which receive additional inputs from the orbitofrontal cortex, medial prefrontal cortex and hippocampus (Price et al., 1987) that enrich encoded stimulus-reinforcer associations with contextual information. Recent neurophysiological evidence in primates suggests that lateral-nucleus neurons encode accurate valuations of particular sensory objects whereas basolateral nucleus are more directly implicated in value comparisons between currently attended objects and decision-making (Grabenhorst et al., 2019; Grabenhorst et al., 2023). The basolateral and basomedial nuclei send direct outputs to the prefrontal cortex and striatum—routes by which the amygdala can influence neural systems associated with decision-making and action selection. A second critical output involves the amygdala’s centromedial nuclei, which process inputs from other amygdala nuclei to regulate behavioral and autonomic responses to affective stimuli via projections to the hypothalamus, basal forebrain, periaqueductal gray, midbrain and brainstem (Price et al., 1987; Price, 2003). Complex inhibitory systems modulate information processing in these amygdala circuits (Duvarci and Pare, 2014).

Its extrinsic and elaborate intrinsic connections (Pitkanen et al., 1997; Pitkanen and Amaral, 1998) enable the amygdala to process the value of sensory stimuli based on learned stimulus-reinforcer associations, and integrate this information with current contexts, memories, and internal states to regulate emotion, attention, memory, physiological and behavioral responses (Rolls, 2000; Paton et al., 2006; Murray, 2007; Johansen et al., 2011; Duvarci and Pare, 2014; Saez et al., 2015; Gothard, 2020; Grabenhorst and Schultz, 2021). The amygdala is a complex structure composed of different subregions with many different cell types. Accordingly, its neurons have been shown to be related to a diversity of functions including not only value-coding and decision-making (Paton et al., 2006; Grabenhorst et al., 2012; Chang et al., 2015; Hernadi et al., 2015; Grabenhorst et al., 2016; Costa et al., 2019; Grabenhorst et al., 2019; Jezzini and Padoa-Schioppa, 2020; Grabenhorst et al., 2023), but also the distinct processing of stimulus intensity, salience, arousal and other processes important in emotion, motivation, and value-guided behavior (Nishijo et al., 2008; Peck et al., 2013; Leathers and Olson, 2017; Iwaoki and Nakamura, 2022; Tang et al., 2024).

As reviewed in detail below, recent evidence indicates that primate amygdala neurons are involved in the component processes underlying the pursuit of future rewards (Grabenhorst et al., 2012; Hernadi et al., 2015; Grabenhorst et al., 2016; Zangemeister et al., 2016). Importantly, the amygdala is also implicated in specific psychiatric conditions and mental-health impairments, including anxiety and depression (Price and Drevets, 2012; Bernardi and Salzman, 2019; Andrews et al., 2022; Klein-Flugge et al., 2022; Fox and Shackman, 2024). Thus, the same neural mechanisms that support successful reward pursuit may also contribute to impaired cognition and emotion in psychiatric conditions in which these processes are dysfunctional.

## Psychological principles underlying the pursuit of future rewards: distinct phases of goal formation and goal pursuit, and the subjective valuation of rewards and costs

Psychological concepts identify component processes underlying the pursuit of future rewards that might be implemented in neural circuits. Goals can be conceptualized as internal representations of desired future states, outcomes or events (Austin and Vancouver, 1996; Brandstatter and Hennecke, 2018). They serve to direct behavior purposefully toward these desired states, inform the selection of behavioral plans for goal-achievement, and regulate attention, effort, and perseverance during goal pursuit (Brandstatter and Hennecke, 2018). Although these concepts stem primarily from human psychology, they can be defined in simple, concrete terms to help operationalization for behavioral and neurophysiological experiments. In the present review, we use the term ‘goal’ to refer specifically to the representation of a future reward that is selected based on subjective valuation of the reward’s attributes and associated costs (i.e., delay, effort). We use the term ‘plan’ to refer to the behavioral means for pursuing and obtaining a goal, for example through a sequence of choices and the actions that execute these choices.

One key principle in psychological models of planned behavior is the distinction between the initial process of goal formation and subsequent goal pursuit (Miller et al., 1960; Berkman and Lieberman, 2009; Bargh et al., 2010; Brandstatter and Hennecke, 2018). For example, economic saving is an elaborate planned behavior that involves forming a self-defined goal for a future reward followed by dynamic, sequential decision-making to achieve the goal (Prelec and Loewenstein, 1998; Benhabib and Bisin, 2005). As we discuss below, neural data suggest that these component processes are encoded as partly distinct activity patterns in primate amygdala neurons.

A second key principle is the initiation of a valuation process that assigns subjective value to goals based on reward, delay, uncertainty, effort cost, and other factors (Sutton and Barto, 1998; Benhabib and Bisin, 2005; Brandstatter and Hennecke, 2018). According to classical expectancy-value theory (Vroom, 1964; Ajzen, 1991; Beckmann and Heckhausen, 2018) the desirability (value) and feasibility (expectancy) of a goal affect goal selection and the effort invested into pursuing the goal. More abstract determinants of goal selection include achievement motives (Brunstein and Heckhausen, 2018), needs for autonomy, competence, and belonging (Deci and Ryan, 2000), social influences (Aarts et al., 2004; Weiss et al., 2023), and external reward (Vroom, 1964). In this review, we focus on the simple and most direct cost factors delay and effort, which can be directly manipulated in neurophysiological experiments. Before we review how primate amygdala neurons integrate valuations of reward goals with delay and effort costs, we first consider how delay and effort costs are evaluated psychologically. This discussion leads to the identification of ‘temporary preferences’ as an important concept that may help explain failures in the pursuit of future rewards.

## Time-sensitive valuations of delay and effort costs govern the pursuit of future rewards

The most direct cost factor involved in the pursuit of future rewards is delay. Delay discounting, i.e., the subjective devaluation of a reward as a function of time until the reward can be obtained, has been behaviorally demonstrated in humans, monkeys and other species (Ainslie, 1974; Ainslie and Herrnstein, 1981; Mazur, 1998; McClure et al., 2004; Kable and Glimcher, 2007; Kobayashi and Schultz, 2008). Consistent with the aforementioned relevance of future rewards to health and wellbeing, dysfunctional delay discounting has been proposed as a ‘trans-disease process’ that may contribute to a range of disorders (Bickel et al., 2012). Indeed, higher rates of delay discounting have been linked to behavioral problems such as obesity, smoking, alcoholism, drug abuse, risky sexual behavior, and gambling problems (Bickel et al., 2012; Scholten et al., 2019).

Delays do not only affect the valuation of future rewards but also the valuation of future costs associated with rewards. For example, in a recent study (Stillman and Woolley, 2023), participants chose between future delivery of 24 cookies or a non-food item (a fashionable bag) matched in delay and monetary value when participants were cued to consider either potential long-term costs of eating cookies (e.g., developing diabetes, heart disease, and obesity) or short-term costs (e.g., feeling nauseous or jittery after eating sugar). Despite long-term costs being more severe, participants cued with short-term costs were significantly less likely to choose the cookies compared to those cued with long-term costs. Thus, the delay of future rewards and costs can affect the selection of reward goals, and this process can be influenced by interventions cueing particular cost types.

Humans and other animals can behave in ways that reflect valuations of reward, delay or effort that are time-sensitive, i.e., valuations that depend on when in time these outcomes will occur. In describing how decisions should be made by rational agents, normative decision theory posits that this time-dependence in reward and cost valuation is irrational and violates a principle known as dynamic consistency (Ainslie and Herrnstein, 1981; Barkan and Busemeyer, 1999). The principle of dynamic consistency states that individuals deciding rationally have consistent preferences throughout time. Therefore, a dynamically consistent decision maker who prefers A to B at a certain point in time will prefer A to B at all other points in time, and their preferences can be modeled with an exponential function that assumes constant rates of discounting, and thus consistent preferences, over time. However, delay discounting has been shown to be modeled more accurately by a nonexponential, hyperbolic function that implements a change in discount rate and can thus accounts for dynamic inconsistency (Green and Myerson, 1996). In accounting for dynamic inconsistency, a hyperbolic discounting function predicts that a decision-maker may have temporary preferences for inferior choice options (Ainslie, 2005). For example, if delay between rewards is discounted more when a smaller, sooner reward is immediate, then as the rewards draw nearer in time the decision-maker will be more likely to prefer the smaller, sooner reward. Temporary preferences were first observed in animal experiments on delay discounting (Rachlin and Green, 1972; Ainslie and Herrnstein, 1981; Mazur, 1998). In a classical delay discounting paradigm in pigeons (Ainslie, 1974), when the delay between smaller, sooner and larger, later rewards was fixed and both rewards were further delayed by equal amounts, the pigeons became less likely to choose the smaller, sooner reward. These reversals of preferences demonstrated that delay-discounting rates can depend on where a delay is placed in time.

Temporary preferences have also been shown in human experiments (Benzion et al., 1989; Green et al., 1994; Kirby and Herrnstein, 1995), which demonstrated how the gap between people’s intentions (future preference) and actions (present-moment preference) may lead to inferior choices. One study (Read and van Leeuwen, 1998) found that participants were more likely to choose an unhealthy compared to a healthy snack when deciding what they would eat immediately compared to deciding what they would eat in 1 week’s time. Similarly, meta-analysis (Sheeran, 2002) highlights the prevalence of this ‘intention-action gap’ related to temporary preferences across a range of health-related behaviors including exercise, contraceptive use, and cancer screening. Across six studies, the median percentage of individuals who set an intention to execute these behaviors yet failed to do so was 47%. Evidently, people can have an initial hope to pursue valuable distant rewards instead of immediately gratifying rewards, but as the competing options of these distant rewards draw closer in time, an increase in the discounting rate of cost factors such as effort and delay causes the inferior alternative to become preferable. Accordingly, temporary preferences resulting from dynamic inconsistency can create a gap between intention and action, undermining an original goal.

Dynamic inconsistency occurs not only in regard to delay discounting but also in regard to effort discounting, i.e., the anticipation of exerted physical or mental effort. Exertion of effort in pursuit of future rewards may come in the form of cognitive effort, as in the mental work required to solve computational problems (Murawski and Bossaerts, 2016; Hong and Stauffer, 2023) or plan a healthy, nutrient-balanced meal, or in the form of physical effort (Hosokawa et al., 2013; Burrell et al., 2023), as in the completion of strenuous exercise to burn calories. Though humans are often averse to effort when anticipating it in the future, effort that has already been expended on a reward can have the opposite effect, increasing subjective value. For example, in the ‘IKEA effect’, having to exert effort assembling a product oneself increases one’s subjective valuation of that item (note that this effect is distinct from the sunk cost fallacy, which involves irrational persistence in a previously chosen option without necessarily valuing it more) (Norton et al., 2012). Thus, the impact of effort on subjective value may depend on when it occurs in time (Inzlicht et al., 2018).

**Figure 2:**
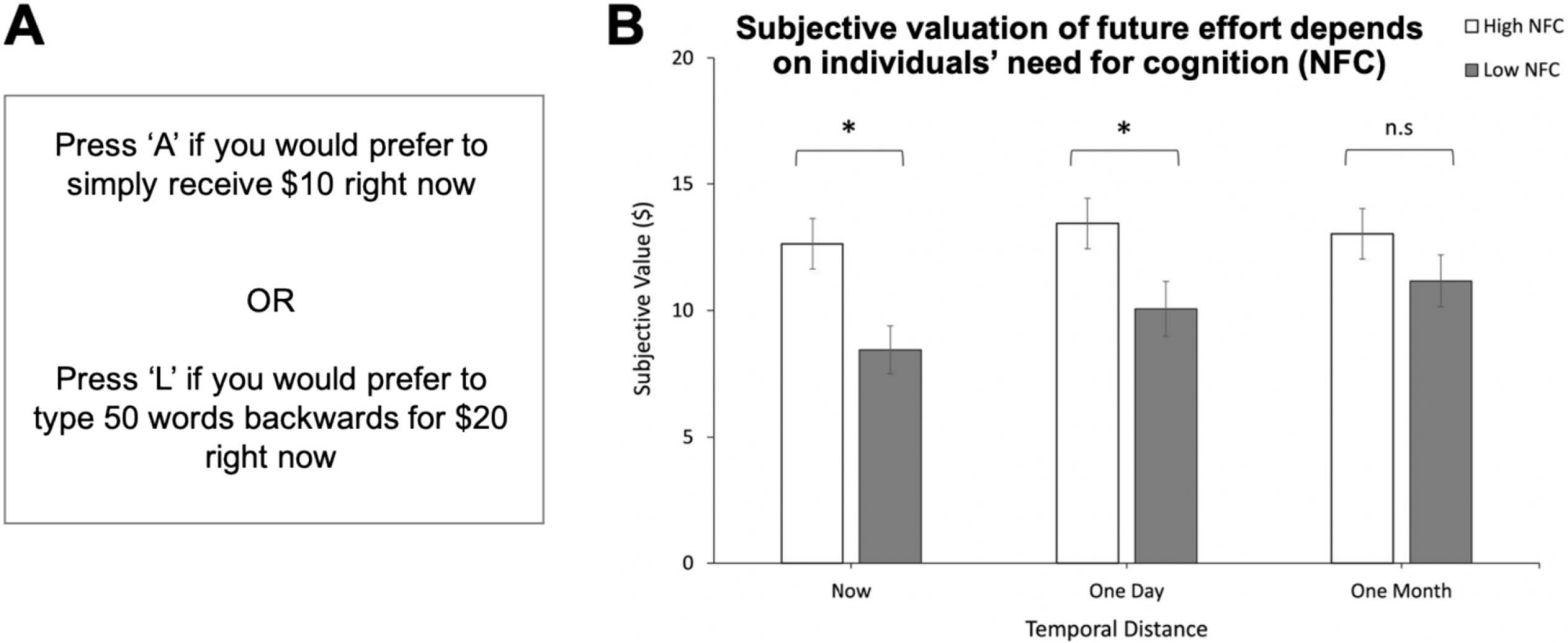
Time-sensitive valuation of cognitive effort: Pushing cognitive effort into the future reduces effort discounting. **(A)** Participants were asked to choose between receiving a smaller reward for no effort or performing the effortful task of typing words backwards at varying temporal delays to receive a larger reward. Repeated choices between options of varying reward size resulted in subjective-value estimates from empirically identified indifference points. **(B)** Individuals low in need for cognition (NFC) displayed dynamic inconsistency in effort-related decision-making. Subjective value of the larger, more effortful reward rose as temporal distance to the anticipated effort increased. Mean subjective values (of an objective $20) are displayed. Error bars: standard errors. *p < 0.05 Adapted with permission from Johnson and Most (2023).

Johnson and Most (2023) investigated whether discounting cognitive effort to obtain a reward depended on how soon the effort occurred in time. They gave individuals the option to receive a smaller reward for no effort or to perform the effortful task of typing words backwards to receive a larger reward (Figure 2A). Participants were informed that each choice was potentially real and they may be rewarded with a shopping voucher of an equivalent monetary value to their choice if they were willing to perform the associated backwards-typing task. Immediacy of the effort was varied by asking participants to imagine exerting varying levels of effort either immediately, in a day, or in a month. The authors observed that individuals tended to discount the effort less when they were making the decision for their future self, compared to making decisions that were immediately relevant (Figure 2B) (Johnson and Most, 2023). Thus, people exhibit dynamic inconsistency in making decisions to obtain rewards by exerting effort, such that the degree of effort discounting depends on how soon the effort occurs. Notably, this effect was only found in individuals low in ‘need for cognition’, a personality trait describing how much one generally enjoys exerting cognitive effort, which suggests that enjoyment of effort may ‘buffer’ against the tendency to be dynamically inconsistent.

Identifying instances of dynamic inconsistency could inform strategies that support goal pursuit. In recognizing the possibility of a temporary preference, an individual setting an intention may choose to implement strategies that minimize future temptations (i.e., avoiding smaller, sooner or smaller, easier rewards) and thus reduce the likelihood of the temporary preference. For example, an individual who intends to exercise in the morning may place their phone out of reach before sleeping to reduce the likelihood of being tempted to stay in bed and sleep in. Remarkably, the aforementioned experiment by Ainslie (1974) found that even pigeons may use such pre-commitment strategies to maximize reward. In a delay discounting paradigm, three out of 10 pigeons consistently pecked a different colored key that prevented the smaller, sooner reward from being offered to them, thus pre-committing to the larger, later reward before the temporary preference arose.

The above findings show that the dynamic, time-sensitive valuation and re-valuation of reward, delay and (physical and cognitive) effort is a central process underlying the pursuit of future rewards that gives rise to temporary preferences. Accordingly, the neural mechanisms involved in selecting future reward goals and the plans to obtain them should reflect the subjective valuations of reward with associated delay and effort costs and also the updating of this integrated signal to account for temporary preferences. Evidence supporting this proposal is reviewed in the next sections and summarized in see Table 1.

## A behavioral paradigm to study the neurophysiology underlying the pursuit of future reward goals: economic reward-saving decisions

Some of the psychological processes reviewed above can be studied in behavioral tasks involving sequential save-spend decisions leading to future rewards. Using this paradigm, neurophysiological studies in monkeys uncovered some of the building blocks underlying the pursuit of future rewards in amygdala neurons (Grabenhorst et al., 2012; Hernadi et al., 2015; Grabenhorst et al., 2016). A subsequent study translated this approach to human functional neuroimaging (Zangemeister et al., 2016, 2019). The behavioral task used in these studies, described next, modeled key aspects of planned, goal-directed behavior: (1) forming a self-determined internal goal based on subjective reward-cost valuations, (2) defining a plan for obtaining the goal, (3) executing the plan by stepwise decision-making and progress-tracking.

**Figure 3:**
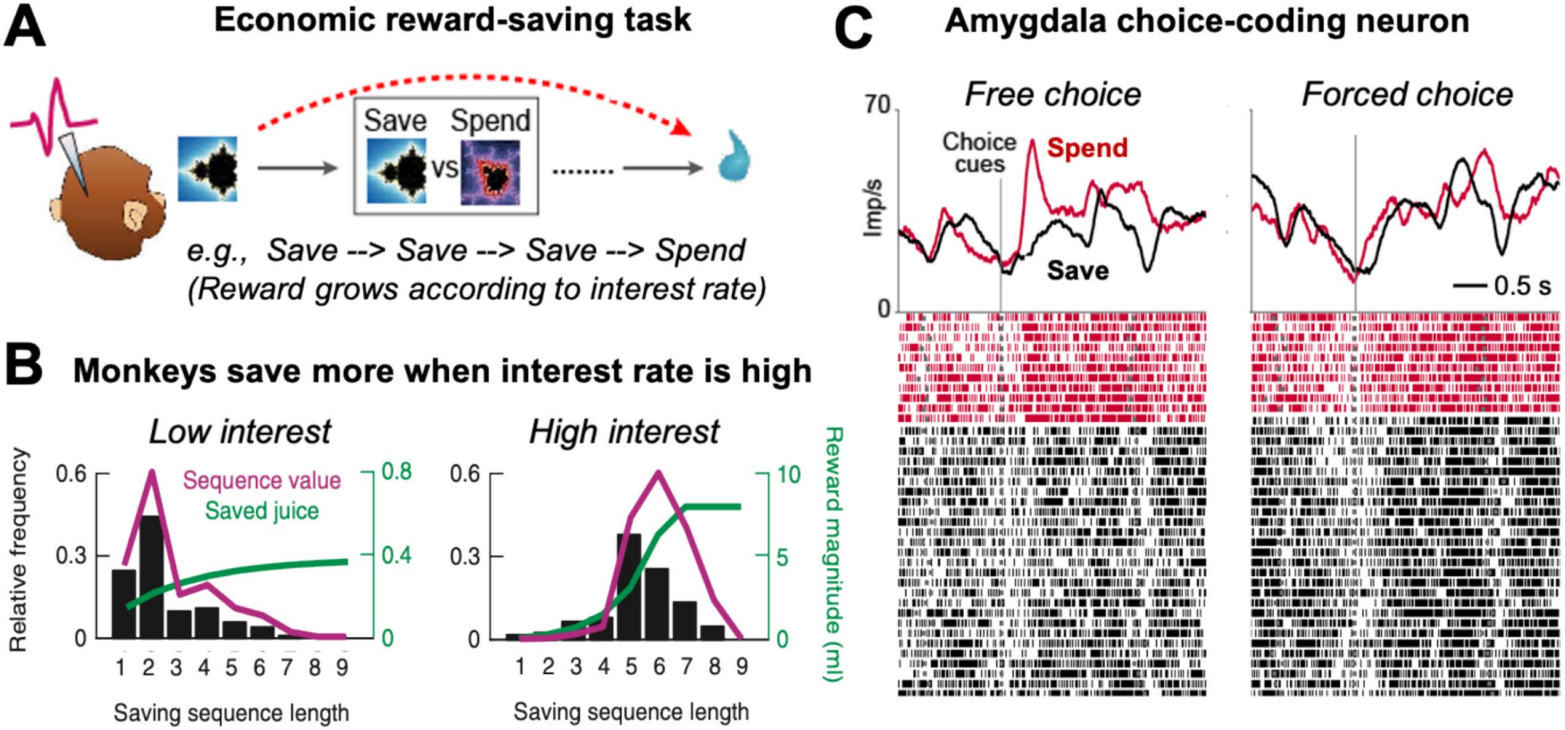
Economic reward-saving behavior in monkeys. **(A)** Schematic of the save-spend task. Monkeys made sequences of save-spend choices to save (i.e., accumulate) liquid reward for later until deciding to spend (i.e., consume) it. The task allowed the monkeys to form an internal goal to obtain a specific future reward and plan to obtain this goal by making a save-spend choice sequence of a specific length. **(B)** Behavior in the saving task. Monkeys produced longer saving sequences, shown by their choice probability for different sequence lengths (black bars), when reward grew exponentially (green curve; reward growth was governed by a cued interest rate). Magenta curve: Subjective value of a saving sequence of defined length estimated from choice probability incorporating reward amount, delay and effort costs. **(C)** Activity of a single amygdala neuron recorded in the saving task (Imp/s: neuronal response measured in impulses per second; raster plot: each line represents a recorded action potential). The neuron responded more strongly at the time of choice when the monkey was going to make a spend choice compared to a save choice on the current trial (left panel). The choice-predictive activity was specific to the free-choice task and disappeared in the instructed, forced-choice control task (right panel), confirming the activity reflected an internally generated choice rather than reward expectation. Adapted with permission from Grabenhorst et al. (2012) and Hernadi et al. (2015).

In the ‘save-spend task’ (Grabenhorst et al., 2012; Hernadi et al., 2015), monkeys made consecutive choices to save (i.e., accumulate) liquid rewards for future trials until they decided to spend (i.e., consume) the saved reward amount (Figure 3A). Save-spend choice options were cued with pre-trained visual conditioned stimuli on a computer monitor. Different save cues indicated different interest rates that governed differential increases in saved reward amounts over consecutive save choices, with high interest rates leading to exponential reward growths (Figure 3B, green curves). The behavioral choice-patterns of the monkeys in this task reflected accurate understanding of the task structure: the animals produced longer sequences of save choices when interest rates were high, and shorter sequences when interest rates were low (Figure 3B, black bars). Control tests confirmed that the monkeys adjusted their behavior even to uncued changes in interest rate and that they tracked saved reward amounts over consecutive trials. The monkeys’ saving behavior was therefore adaptive and internally controlled rather than reflecting fixed, conditioned responses to pretrained cues. Analysis of reaction times showed that responses early on in saving sequences predicted the final length of the saving sequence and the amount of the final saved reward. Thus, the animals anticipated final reward amounts, as early as on the first trial of a saving sequence, consistent with planned behavior directed at internally set reward goals.

An important feature of the task was that it allowed the animals to plan their choices multiple steps in advance, to obtain specific reward amounts through saving sequences of defined lengths requiring particular delay (i.e., waiting times) and effort (i.e., number of operand responses). Accordingly, the subjective value of a reward goal depended not only on final reward amounts but also on costs related to sequence length: because larger reward amounts typically required longer saving sequences, their value was diminished by temporal delay and physical effort. These effects on subjective values were modeled by deriving value from observed behavioral choices for different sequence lengths (Figure 3B, black bars). These ‘sequence value’ functions typically increased with sequence length up to a peak and then decreased with longer sequences that the animal chose less frequently, likely owing to temporal discounting and physical-effort costs (Figure 3B, right panel, magenta curve). This nonlinearity in subjective-value functions made it possible to determine whether neuronal activities encoded subjective value or objective sequence length, as described below.

We note that this approach to reward-saving decisions shares features with ‘token economies’ (Hackenberg, 2009), in which animals earn and accumulate conditioned stimuli and exchange them against different food and liquid reward according to a defined schedule. Similar to goal-directed sequences of save-spend choices described above, token schedules can organize an animal’s behavior over extended time periods that lead to a final (‘terminal’) primary reward. For example, pigeons’ produce sequential responses to accumulate tokens before making a different response to exchange them against food or liquid reward (Yankelevitz et al., 2008; DeFulio et al., 2014). Similarly, monkeys anticipate rewards in instructed sequential reward-schedule tasks with visual cues indicating reward proximity (Shidara and Richmond, 2002; Sugase-Miyamoto and Richmond, 2005) and make reward-maximizing choices using tokens that signify gain or loss of primary reinforcers (Yang et al., 2022; Tang et al., 2024).

## Primate amygdala neurons encode plans for future rewards: the value of goals, behavioral plans for obtaining them, and adaptive progress during goal pursuit

The primate amygdala has long been implicated in reward-guided behavior (Rolls, 2000; Baxter and Murray, 2002; Paton et al., 2006; Belova et al., 2007; Murray, 2007) but only recently has been shown to participate in value-based decision-making (Grabenhorst et al., 2012; Rudebeck et al., 2013; Rudebeck et al., 2017; Costa et al., 2019; Grabenhorst et al., 2019; Jezzini and Padoa-Schioppa, 2020; Grabenhorst et al., 2023) and the planned pursuit of future reward goals (Grabenhorst et al., 2012; Hernadi et al., 2015; Grabenhorst et al., 2016; Zangemeister et al., 2016). During performance of the save-spend task introduced in the previous section, single-cell recordings showed that a significant number of amygdala neurons exhibited activity patterns that predicted the monkeys’ save-spend choice on individual trials (Grabenhorst et al., 2012). These choice-predictive activities (Figure 3C) were not explained by left–right actions, nor did they reflect subjective values of save-spend choices, which were encoded separately. Rather, these amygdala neurons signaled the monkey’s economic choice to either spend reward for immediate consumption or save it for the future. Choice-predictive activities were in most cases specific to freely made choices, as they disappeared in a control task involving forced, instructed save-spend choices (Figure 3C, right panel). Thus, amygdala neurons predicted the monkey’s self-determined choices to either pursue a future goal by saving reward for later or to spend the accumulated reward immediately. More recent studies implicated amygdala neurons in choices for different types of reward (Jezzini and Padoa-Schioppa, 2020), in explore-exploit decisions (Costa et al., 2019), social decisions (Chang et al., 2015; Grabenhorst et al., 2019), and in decision computations that compare currently viewed and recently viewed choice options (Grabenhorst et al., 2023).

**Figure 4:**
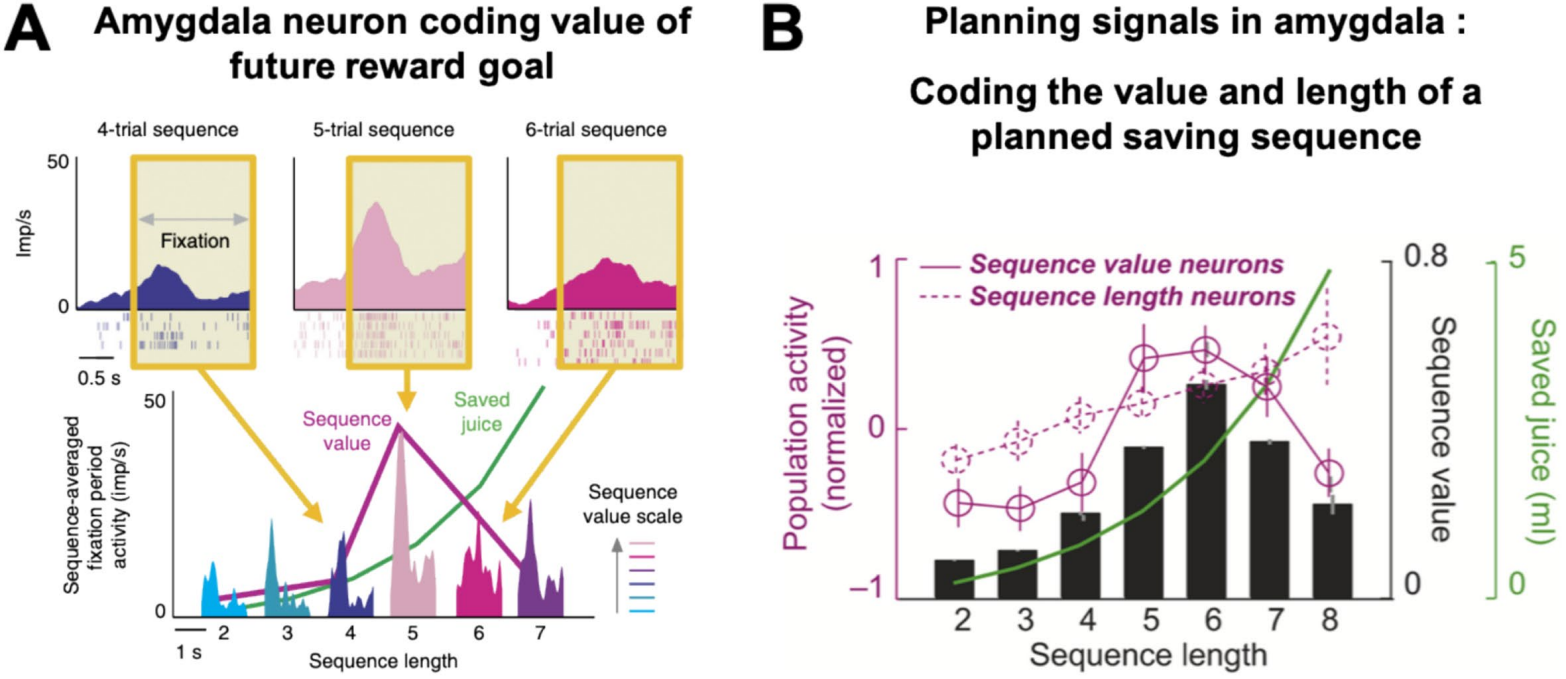
Amygdala neurons encode value and length of monkeys’ saving plans. **(A)** An amygdala neuron with prospective activity that reflected the subjective value of the monkey’s internal saving plan. The neuron’s activity depended on the subjective value of the current sequence (‘sequence value’) that would only be completed several moments into the future. Top: Activity at trial start (yellow area) was highest for the sequence in which the monkey would eventually spend on the fifth trial, as this sequence had the highest subjective value. Bottom: activity averages for all sequence lengths (for example, the light pink activation represents the mean trial-start activity for all five-trial sequences, averaged over trials 1 to 5). Activity reflected sequence value (magenta curve), rather than linear sequence length or objective reward amount (green curve). **(B)** Amygdala neurons with prospective activity for future rewards. Neurons signaled the length of the planned choice sequence (dashed magenta curve, population activity, *N* = 92 neurons) or its subjective value (solid magenta curve, *N* = 93 neurons). Value activity was highest during sequences lasting six trials, which had the highest subjective value (black bars), i.e., these sequences were typically preferred by the animals, because they offered large reward (green curve) for moderate delay and physical effort. Adapted with permission from Hernadi et al. (2015).

Amygdala neurons did not simply encode save-spend choices related to single trials but also showed more complex, prospective activities that were critical for optimal task performance (Hernadi et al., 2015; Grabenhorst et al., 2016). These ‘planning activities’ preceded the end of a saving sequence by several steps and encoded information about the final reward goal, which was self-determined by the monkey and existed only internally at the time of saving. Specifically, amygdala planning activities signaled two well-defined features of the animal’s internal saving plan (Hernadi et al., 2015): some neurons encoded ‘sequence value’, defined as the subjective value of the current saving sequence (Figures 4A,B, solid magenta curve), while a separate set of neurons encoded the ‘sequence length’, defined by the number of planned, forthcoming save choices (Figure 4B, dashed magenta curve). For example, the neuron in Figure 4A had recurring phasic responses on each trial that were highest during sequences in which the monkey would eventually spend on the fifth trial and lower for shorter or longer sequences. This activity profile reflected the distribution of subjective values derived from the monkey’s choice preferences: five-trial sequences had the highest value under the current interest rate, as the animal chose them most frequently. Importantly, subjective value was a non-monotonic function of sequence length: depending on the current interest rate, subjective value was highest for intermediate sequence lengths (Figure 4B, black bars) that offered a compromise between large rewards (Figure 4B, green curve) and moderate delays and effort. These planning activities typically disappeared during instructed trials, despite comparable reward timing and anticipation, and in control analyses were shown to be unrelated to reward proximity and expectation.

Thus, amygdala neurons in the save-spend task encoded two key components of the monkeys’ internal saving plan: the subjective value of the current reward goal (‘sequence value’) and the behavioral plan to achieve the goal through a choice sequence of defined length (‘sequence length’).

**Figure 5:**
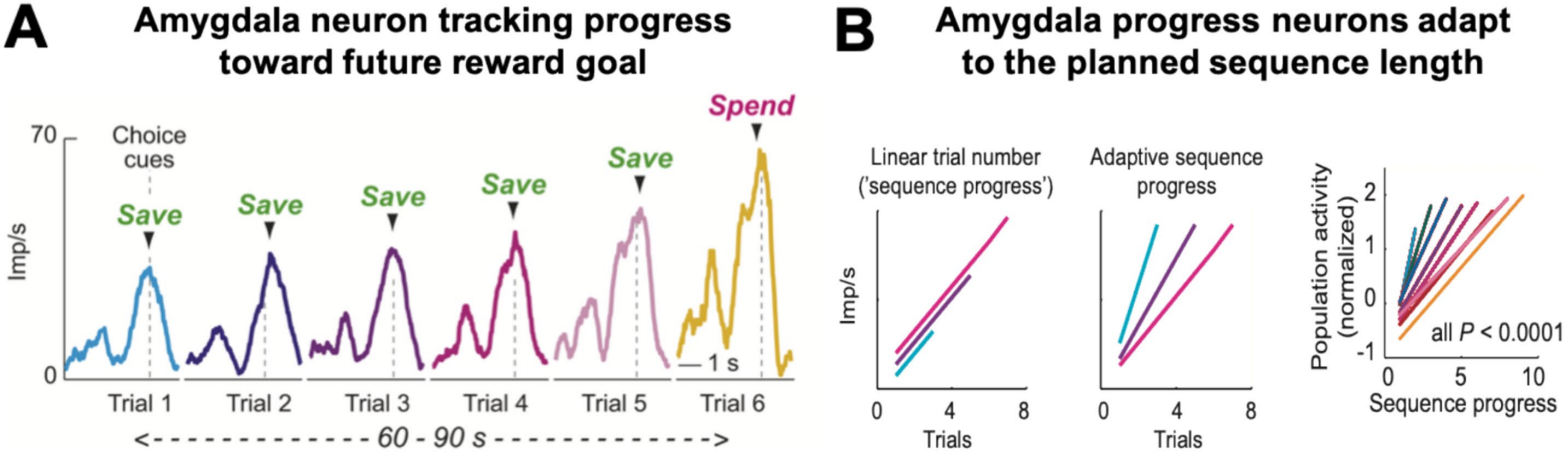
Progress-tracking in amygdala. **(A)** Ramping activity of an amygdala neuron in a saving sequence lasting six trials. The neuron’s responses at the time of choice increased with each consecutive step in the saving sequence. **(B)** The slope of the neuronal ramping activity in amygdala for different sequences (right panel) adapted to the final sequence length (‘adaptive sequence progress’, middle schematic); it did not increase linearly with elapsed time or trial number (left schematic), consistent with progress-tracking rather than time-tracking. Adapted with permission from Grabenhorst et al. (2016).

A distinct, third type of amygdala activity signaled the monkeys’ progress over the course of a saving sequence (Grabenhorst et al., 2016). These progress-tracking neurons showed gradually increasing, ‘ramping’ activity over consecutive save choices until the monkey decided to spend the saved reward (Figure 5A). Importantly, the responses occurred in the absence of external progress cues and were often specific to internally guided choices. Moreover, the slope of this ramping activity depended on the forthcoming sequence length, with steeper neuronal ramping for shorter sequences (Figure 5B). These findings suggest that amygdala progress-tracking signals adapt to the monkey’s internal plan to execute a specific sequence length. Such continual evaluation of progress by amygdala neurons seems crucial for aligning choices with internal plans and for successful goal pursuit (Johnson and Busemeyer, 2001; Benhabib and Bisin, 2005; Berkman and Lieberman, 2009).

Taken together, different neurons in the primate amygdala encode important building blocks for the pursuit of future rewards: (1) the subjective value of the current reward goal (‘sequence value’), (2) the behavioral saving plan for achieving the goal (‘sequence length), (3) the save-spend choices that realized this plan at specific choice points, and (4) the internally tracked progress toward the reward goal. In relation to the psychological concepts of goal pursuit discussed above, it is possible that variation in these amygdala signals within and across individuals might underlie individual differences in the capacity to pursue long-term rewards (see Table 1). For example, fluctuations in the amygdala’s coding of current reward goals might lead to dynamic revaluation of the chosen goal and its alternatives at critical choice points, which could provide a neurophysiological mechanism for temporary preferences. Further, the initial goal valuation and goal formation could be impacted by changes in the function of sequence-value neurons: Exaggerated valuation of a goal’s rewarding aspects or diminished discounting of delay and effort costs could result in neuronal encoding and behavioral pursuit of unrealistic goals. Conversely, diminished valuation of future rewards and exaggerated delay and effort discounting could result in the absence of neuronally encoded future rewards and behavioral amotivation. Importantly, the neuronal signals for sequence progress, sequence length and sequence value described above fluctuated with behavioral performance: they were diminished on error trials, when the animal failed to complete a trial correctly, and subsequently reinstated, suggesting behavioral relevance of these amygdala signals. Thus, the amygdala signals reported above might be behaviorally relevant during an individual’s pursuit of future rewards.

## Future reward goals and planning activity in the human amygdala

**Figure 6:**
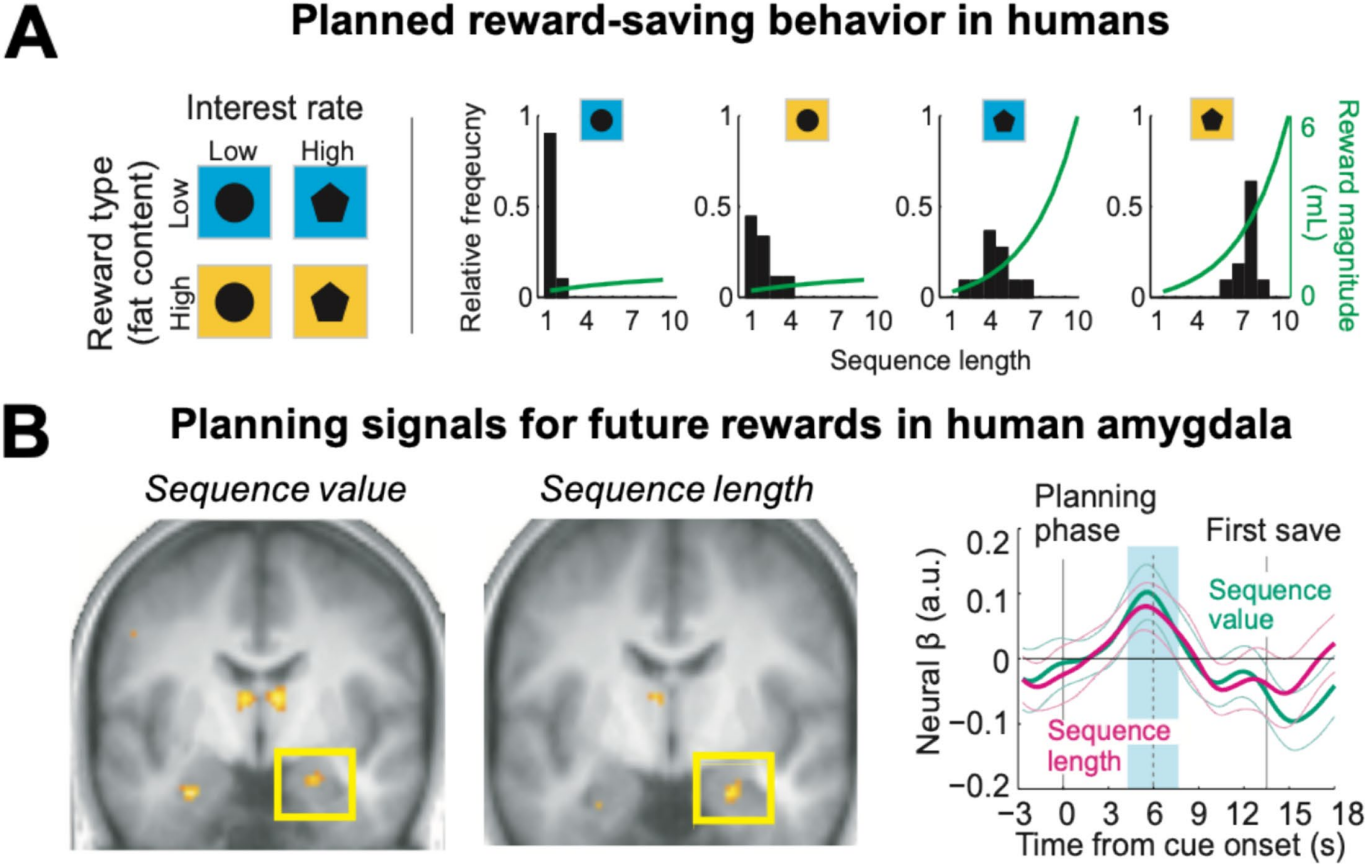
Planning activity for future rewards in the human amygdala. **(A)** Human participants in the MRI scanner performed save-spend choice sequences to obtain liquid rewards (milkshakes) of different sizes under varying interest rates and reward types cued by visual stimuli (left). Participants produced longer saving sequences for higher interest rates and preferred rewards (right). **(B)** The amygdala encoded the subjective value and planned length of the current saving sequence before participants made their first choice. Time-course analyses (right, betas from multiple-regression analysis) confirmed distinct amygdala signals for sequence value and sequence length (blue shading: analysis period). Adapted with permission from Zangemeister et al. (2016).

The monkey single-neuron investigations reviewed above inspired a human functional neuroimaging study that aimed to translate these findings to the human brain (Zangemeister et al., 2016, 2019). Healthy volunteers performed the save-spend task while their brain activity was measured using functional magnetic resonance imaging (fMRI). As in the monkey study, human participants could plan and execute economic choice sequences to drink liquid rewards—delivered in the MRI scanner—that increased in amount according to the current interest rate. The reward also varied in type, consisting either of a low-fat high-sugar milkshake or a typically preferred high-fat high-sugar milkshake (Figure 6A, left panel).

Similar to the monkeys, the human participants produced longer saving sequences when interest rates were high, but also saved longer for the more preferred high-fat rewards, consistent with the assignment of subjective values to different saving goals (Figure 6A, right panel). At the start of a saving sequence, before participants made the first choice to save or spend, neural activity in the amygdala reflected the key planning variables identified in the monkey studies: the subjective value of the planned saving sequence and its length (Figure 6B). Importantly, the degree to which these neural planning activities depended on the current reward type (high vs. low fat) matched individual participants’ reward preferences. Thus, amygdala planning activities incorporate information about reward amount, reward type, temporal delay and physical effort. Whole-brain imaging identified other brain areas that contributed to planned reward-saving decisions: neural activities in medial and dorsolateral prefrontal cortex areas reflected planned sequence length and functional coupling with each other and with the amygdala. A distinct prospective signal recorded in the ventromedial prefrontal cortex reflected participants’ reported saving intentions at the beginning of a new saving sequence (Zangemeister et al., 2019).

Thus, the human amygdala encodes different aspects of economic saving plans as part of a functionally connected frontal-amygdala network. Importantly, planning signals for internally generated reward goals depended not only on reward amount, delay and effort cost but also on the subjective valuation of different reward types. That aspects of the single-cell activity patterns related to goal pursuit originally identified in the monkey amygdala can be measured in humans using fMRI opens up the possibility to investigate how these activities might be altered in psychiatric conditions, and how they could be affected by behavioral interventions. To stimulate research, we include in Table 1 suggestions for how changes in specific amygdala signals could be linked to failures or improvements in the pursuit of long-term goals.

## Neurophysiology of planned behavior beyond amygdala

The prospective amygdala signals reviewed above likely contribute to planned behavior together with activity patterns in other brain areas, briefly reviewed here. Planning functions have traditionally been associated with frontal-lobe regions (Miller et al., 1960; Tanji and Hoshi, 2001; Stuss and Knight, 2002; Passingham and Wise, 2012). In monkeys, single neurons in specific frontal regions including lateral-prefrontal, premotor, supplementary-motor and presupplementary-motor cortices encode planned action sequences and the execution and updating of motor plans (Tanji and Shima, 1994; Shima et al., 1996; Shima and Tanji, 1998; Mushiake et al., 2006; Sohn and Lee, 2007). For example, in a remarkable study (Shima et al., 2007), monkeys performed action sequences involving different combinations of push, turn, and pull movements with a lever. Before the monkeys executed each sequence, lateral prefrontal neurons encoded planned sequences in abstract ‘categories’, i.e., according to whether they required repetition, alternation or pairings of the same action. Complementing the amygdala neurons described above that encode behavioral sequences in terms of their subjective value (Hernadi et al., 2015), these prefrontal neurons could help specify the behavioral means by which future rewards can be pursued. Further, single-cell activities in frontal-lobe areas (motor, premotor, supplementary-motor cortices), the putamen and the striatum also precede self-initiated movements (Okano and Tanji, 1987; Romo and Schultz, 1987; Kurata and Wise, 1988; Schultz and Romo, 1992; Lee et al., 2006), which represent the behavioral means for executing a plan. Further, activities in human medial frontal cortex, monkey striatum and monkey dopamine neurons encode the subjective value of delayed rewards and thereby likely contribute to prospective goal valuations (Kable and Glimcher, 2007; McClure et al., 2007; Kim et al., 2008; Kobayashi and Schultz, 2008). In multistep reinforcement learning, prefrontal-striatal systems in human imaging studies encode evaluation of reward outcomes associated with externally defined choice paths (Wunderlich et al., 2012; Doll et al., 2015). Further, when human decision-makers calibrate their behavioral persistence while waiting for rewards, neural activity in medial prefrontal cortex reflects this context-sensitive subjective valuation (McGuire and Kable, 2015).

Thus, beyond amygdala, neural activity in connected brain areas contributes to internally planned, goal-oriented behaviors. Following earlier proposals (Shima and Tanji, 1998), we suggest that amygdala neurons signaling the subjective value of reward goals and the plans to obtain them (Grabenhorst et al., 2012; Hernadi et al., 2015; Grabenhorst et al., 2016; Zangemeister et al., 2016) could provide value-based inputs to other brain systems to direct planned, self-initiated behavior toward future rewards.

## Amygdala neurons and self-determination

One important aspect of the findings in the save-spend task reviewed above is that amygdala activity related to choices, reward goals and progress-tracking was typically specific to self-determined (rather than externally instructed) saving behavior (Grabenhorst et al., 2012; Hernadi et al., 2015; Grabenhorst et al., 2016; Zangemeister et al., 2016). This observation links the interpretation of the amygdala’s involvement in goal pursuit to psychological theories of self-determined behaviors.

Behaviors generated by oneself differ in important ways from those that are externally imposed, as conceptualized by one of the most influential theories of human motivation: Self-Determination Theory (SDT, Deci and Ryan, 2000). In the terminology of SDT, ‘internally motivated’ behaviors are more likely to be accompanied by experiences of autonomy. In contrast, behaviors driven primarily by social forces are termed ‘externally motivated’ and typically lack the feelings of agency that accompany internally motivated behaviors. Planning signals in the amygdala were shown to depend on whether the behavior was internally or externally motivated, as these signals tended to disappear when the decision maker was instructed by a visual cue which action to take (Grabenhorst et al., 2012; Hernadi et al., 2015; Grabenhorst et al., 2016). Therefore, amygdala neurons seem sensitive to whether choices are self-determined or externally imposed, which may provide a basis for experiencing decisions as autonomous. In the SDT literature, autonomy creates higher levels of behavioral persistence and satisfaction (Grolnick and Ryan, 1987; Vallerand and Bissonnette, 1992; Sheldon and Houser-Marko, 2001). As the amygdala sends projections to brain areas involved in reward-related subjective experiences, including the medial prefrontal cortex and orbitofrontal cortex (Kringelbach et al., 2003; Rolls et al., 2003; Grabenhorst et al., 2007; Rolls and Grabenhorst, 2008; Grabenhorst et al., 2010; Grabenhorst and Rolls, 2011), it is possible that amygdala signals that reflect whether behavior was performed in an autonomous, self-determined manner may contribute to the subjective experience of autonomy. Future research could therefore examine whether amygdala signals related to self-determined decisions contribute to the positive psychological outcomes associated with self-determined behavior. As past research has suggested that promoting experiences of autonomy could mitigate depressive symptoms (Moore et al., 2021), future research could also investigate the role of these amygdala signals in depression and potentially in other psychiatric conditions in which the amygdala is implicated (Price and Drevets, 2012; Bernardi and Salzman, 2019; Andrews et al., 2022; Klein-Flugge et al., 2022; Fox and Shackman, 2024).

## Conclusion and perspective: amygdala neurons as possible targets for behavioral-change interventions

The successful pursuit of future rewards requires the formation of a goal based on subjective reward and cost valuations, the definition of a plan to obtain the goal, and stepwise decision-making and progress-monitoring during goal pursuit. The amygdala signals reviewed above related to the value of internal reward goals and plans for obtaining them seem well-suited to direct the self-determined pursuit of future rewards. As individuals can be inconsistent in their evaluations of cost factors such as effort and delay, they may prefer a distant goal at one point in time but then switch their preference to an inferior but immediately-gratifying reward. These temporary preferences for immediate but inferior rewards are therefore a key concept for explaining impulsive behaviors that reflect time-dependent revaluation of reward goals and cost factors. Thus, future studies could examine whether amygdala neurons encoding the value of distant rewards update their activity to reflect time-dependent revaluations of goals. Such neurons could provide a neurophysiological basis for the emergence of temporary preferences and so-called ‘self-defeating’ behaviors, i.e., a choice made during goal pursuit that deviates from an initially formed goal and prioritizes an inferior reward over a previously stated preference for a larger but distant reward. As indicated above, successes and failures in the pursuit of future reward are highly relevant to human behavior, well-being, and mental health across life domains. A valuable research program into these questions could combine single-cell recordings in monkeys making complex, primate-typical decisions about future rewards with functional neuroimaging in humans performing the same decision tasks. This translational approach could provide evidence on detailed, single-neuron activity patterns underlying reward-pursuit and temporary preferences, and on the related neural correlates in human brain areas and brain systems, which could be used to investigate neural mechanisms of behavioral-change interventions.

Although confirmation of the behavioral relevance of amygdala neurons will require further experiments, we suggest that the amygdala’s activity patterns could represent potential vulnerabilities for dysfunctional reward-guided behavior and related mental-health impairments in conditions in which the amygdala is implicated, including depression, anxiety and addiction (Price and Drevets, 2012; Bernardi and Salzman, 2019; Andrews et al., 2022; Klein-Flugge et al., 2022; Fox and Shackman, 2024). The same neural signals may also be targets for interventions (Gollwitzer and Schaal, 1998; Diamond and Lee, 2011; Belanger-Gravel et al., 2013; Michie et al., 2013; Sheeran et al., 2013; Scholten et al., 2019), in that the effects of interventions aiming for behavioral change, such as mental contrasting (Duckworth et al., 2013), may potentially be mediated by altered activity patterns of these amygdala neurons. In Table 1, we propose some specific ways in which known amygdala signals with particular functions in the pursuit of future rewards could represent vulnerabilities for dysfunction and targets for intervention. These proposals are made with the intention of stimulating further discussion and research, although we acknowledge that these suggestions remain speculative and will await confirmation by behavioral and neurophysiological data. Further to investigating the role of the amygdala in the behavior change strategies described in Table 1, future research could investigate how the complex psychological mechanisms underlying behavioral-change strategies may relate to amygdala function. For example, it has been proposed that one mechanism of mental contrasting is changing how individuals interpret specific situations (Oettingen and Schworer, 2013). After mental contrasting, an individual may begin to interpret an opportunity to eat a new flavor of ice cream as an obstacle to their weight loss progress rather than an exciting new experience. Thus, understanding the functions of amygdala neurons in the pursuit of future rewards may not only address basic biological questions concerning the neural basis of motivated behavior but may also lead to mechanistic explanations for successes and failures of behavior-change interventions.
